# Comparative analysis of *Staphylococcus aureus* and *Escherichia coli* microcalorimetric growth

**DOI:** 10.1186/1471-2180-13-171

**Published:** 2013-07-24

**Authors:** Dragos C Zaharia, Alexandru A Muntean, Mihnea G Popa, Alexandru T Steriade, Octavian Balint, Roxana Micut, Corneliu Iftene, Ioana Tofolean, Vlad T Popa, Cristian Baicus, Miron A Bogdan, Mircea I Popa

**Affiliations:** 1 University of Medicine and Pharmacy, “Carol Davila”, Eroii Sanitari Bulevard 8, Bucharest, Romania; 2 Romanian Academy, Institute of Physical Chemistry, “Ilie Murgulescu”, Splaiul Independentei 202, 060021, Bucharest, Romania

## Abstract

**Background:**

Microcalorimetric bacterial growth studies have illustrated that thermograms differ significantly with both culture media and strain. The present contribution examines the possibility of discriminating between certain bacterial strains by microcalorimetry and the qualitative and quantitative contribution of the sample volume to the observed thermograms. Growth patterns of samples of *Staphylococcus aureus* (ATCC 25923) and *Escherichia coli* (ATCC 25922) were analyzed. Certain features of the thermograms that may serve to distinguish between these bacterial strains were identified.

**Results:**

The thermograms of the two bacterial strains with sample volumes ranging from 0.3 to 0.7 ml and same initial bacterial concentration were analyzed. Both strains exhibit a roughly 2-peak shape that differs by peak amplitude and position along the time scale. Seven parameters corresponding to the thermogram key points related to time and heat flow values were proposed and statistically analyzed. The most relevant parameters appear to be the time to reach a heat flow of 0.05 mW (1.67 ± 0.46 h in *E. coli* vs. 2.99 ± 0.53 h in *S. aureus*, p < 0.0001), the time to reach the first peak (3.84 ± 0.5 h vs. 5.17 ± 0.49 h, p < 0.0001) and the first peak value (0.19 ± 0.02 mW vs. 0.086 ± 0.012 mW, p < 0.0001). The statistical analysis on 4 parameters of volume-normalized heat flow thermograms showed that the time to reach a volume-normalized heat flow of 0.1 mW/ml (1.75 ± 0.37 h in *E. coli* vs. 2.87 ± 0.65 h in *S. aureus*, p < 0.005), the time to reach the first volume-normalized peak (3.78 ± 0.47 h vs. 5.12 ± 0.52 h, p < 0.0001) and the first volume-normalized peak value (0.35 ± 0.05 mW/ml vs. 0.181 ± 0.040 mW/ml, p < 0.0001) seem to be the most relevant. Peakfit® decomposition and analysis of the observed thermograms complements the statistical analysis via quantitative arguments, indicating that: (1) the first peak pertains to a faster, “dissolved oxygen” bacterial growth (where the dissolved oxygen in the initial suspension acts as a limiting factor); (2) the second peak indicates a slower “diffused oxygen” growth that involves transport of oxygen contained in the unfilled part of the microcalorimetric cell; (3) a strictly fermentative growth component may slightly contribute to the observed complex thermal signal.

**Conclusion:**

The investigated strains of *Staphylococcus aureus* and *Escherichia coli* display, under similar experimental conditions, distinct thermal growth patterns. The two strains can be easily differentiated using a selection of the proposed parameters. The presented Peakfit analysis of the complex thermal signal provides the necessary means for establishing the optimal growth conditions of various bacterial strains. These conditions are needed for the standardization of the isothermal microcalorimetry method in view of its further use in qualitative and quantitative estimation of bacterial growth.

## Background

Among the wide range of microcalorimetry applications, an important and promising one is the direct measurement of heat generated by the biological processes within living cells. Microorganisms (including bacteria) are reported to produce heat to an average of 1–3 pW per cell [1].

The bacterial replication process can be monitored in real time due to the heat production associated with their metabolic activity recorded as heat flow versus time. Modern isothermal microcalorimeters (IMC) allow for the detection of less than one microwatt in power change. As a result, as few as 10,000-100,000 active bacterial cells in a culture are sufficient to produce a real-time signal, dynamically related to the number of cells present and their activity [1]. For aerobic growth, a recent contribution [2] used an extension of the above range to 1-4 pW per cell based on earlier reported results [3], thus pointing to a range of calorimetric detection of 6250 – 25000 cells per ml. Therefore, microcalorimetry may be considered as one of the most sensitive tools in the study of bacterial growth.

Recent microcalorimetric studies regarding the antibacterial effect or interaction of different compounds (chemical or biological) with certain bacterial strains further acknowledged the reliability and utility of this method [4-6].

In our previous contribution, we have proved that the thermal growth signal obtained via IMC is reproducible within certain experimental conditions (temperature, bacterial concentration, sample thermal history) [7].

Observations from classical microbiology cultures have shown that bacterial metabolism varies by strain, a feature widely used in bacterial identification. Although reliable and extremely useful in the clinical environment, bacterial identification by classical biochemical tests and by more modern Analytical Profile Index (API - Biomérieux) batteries can take several days. Different metabolic profiles of bacteria should be expressed in different microcalorimetric growth patterns (thermograms). In our past experience we noticed significant differences in thermograms of various bacterial strains. The analysis of real time thermal growth patterns [8] revealed significant differences in less than 8 hours. In principle, rapid strains discrimination by thermal signal analysis is thus feasible. In terms of rapidity and descriptive information, microcalorimetry could complement other modern rapid bacterial identification and characterization techniques such as 16S ribosomal DNA sequencing [9], commercial systems such as Vitek® [10] from Biomérieux and matrix-assisted laser desorption/ionization time-of-flight mass spectrometry (MALDI-TOF) [11].

In the present contribution, the differences in microcalorimetric growth patterns obtained from two distinct bacterial strains of *Staphylococcus aureus* and *Escherichia coli* were analyzed*.* To this purpose we have studied samples kept in cold storage, proven to yield better microcalorimetric reproducibility when working with single channel calorimeters, as shown in our previous paper [7]. Moreover, the present research aims to illustrate the most relevant parameters that can be used for the systematic classification of the growth patterns. We emphasize that bacterial strains that make the object of present experiments (*Staphylococcus aureus* and *Escherichia coli*) are known to grow in both aerobic and anaerobic conditions [12,13]. Apart from describing the differences in bacterial thermograms, factors that influence the results were also analyzed (oxygen availability and metabolism and time spent in cold storage).

## Results and discussion

A series of 18 *Escherichia coli* and 8 *Staphylococcus aureus* experiments with samples of different volumes (0.3, 0.4, 0.5, 0.6, 0.7 ml) were analyzed. All experiments used the same bacterial concentration and culture medium. All experiments displayed complex thermal signals. Qualitative (section A) and quantitative (section B) assessments of the thermograms of the two bacterial strains were carried out. To better understand the influence of experimental conditions (oxygen availability and metabolism, time spent in cold storage) on the reported results, additional experiments were devised using physiological saline and mineral (paraffin) oil (section C). For the present stage of analysis, the number of distinctive thermal growth features taken into account was restricted to a minimum.

### Qualitative analysis

**Figure 1:**
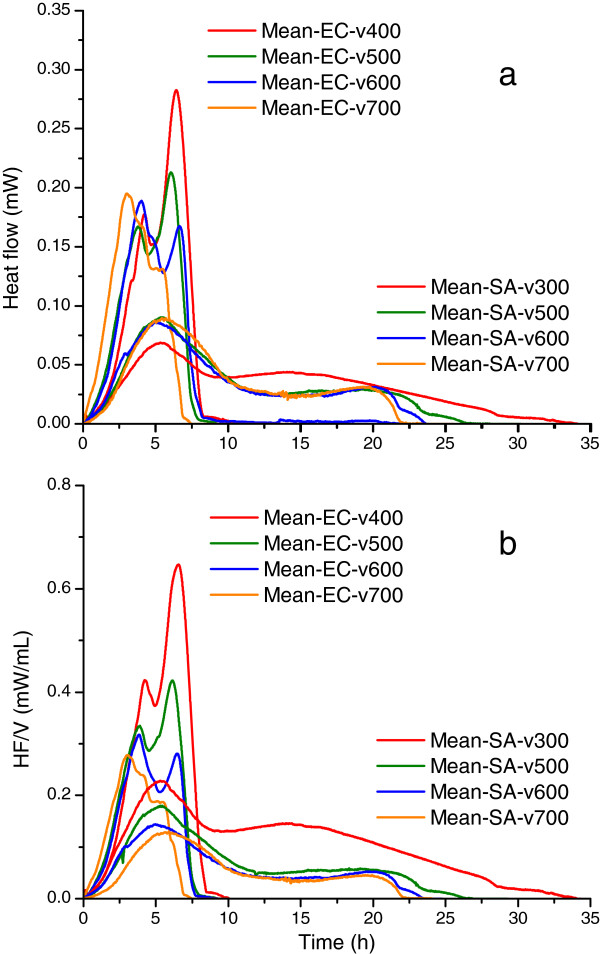
**Mean thermograms of *Escherichia coli *and *Staphylococcus aureus *for samples with different volumes. a**. Mean thermograms of *Escherichia coli* (n = 18) and *Staphylococcus aureus* (n = 8) at various volumes of bacterial suspension. The mean thermograms were obtained averaging the same volume sample runs. Both species exhibit a double-peak behavior but with sizable shape differences. EC - *Escherichia coli*, SA - *Staphylococcus aureus*. **b**. Mean volume-normalized thermograms (expressed as mW/ml bacterial suspension) of *Escherichia coli* and *Staphylococcus aureus* generated using the Calisto software (HF/V: heat flow/sample volume). The legends display sample volume in microliters.

As illustrated in Figure 1a, microcalorimetric growth data of the two bacterial strains display a major similarity, as well as several differences between the thermograms, and these findings are valid for the entire range of sample volumes utilized.

#### Similarity

All recorded thermograms display a 2-peak shape of the thermal signal, for both strains. The sizes of these two peaks exhibit an opposite behavior: the first one increases, while the second one decreases with increase of the sample volume (more evident in the *E. coli* strain thermograms, Figure 1a).

#### Differences

The *E. coli* growth thermograms extend over a total time of around 10–12 hours, significantly less than those of *S. aureus* (20–30 hours). The heat flow amplitude obtained for *Escherichia coli* is much higher than the corresponding one of *Staphylococcus aureus* (around 0.20 mW vs. 0.075 mW). Furthermore, the second peak of *S. aureus* is much broader. The time needed to detect the thermal signal attributed to bacterial growth is lower in the case of the *E. coli* (i.e. the thermal expression of growth is faster). These qualitative observations were validated by quantitative analysis of the thermograms, with the aim to identify reliable parameters that can be used for fast and efficient calorimetric discrimination of the bacterial strains.

### Quantitative analysis

By analogy with the terminology of Monod [14] the total thermal effect calculated from the observed thermogram was termed “total thermal growth”. This quantity may be expressed as the absolute (J) or specific (J/g or J/ml suspension) value.

#### Similarity

**Figure 2:**
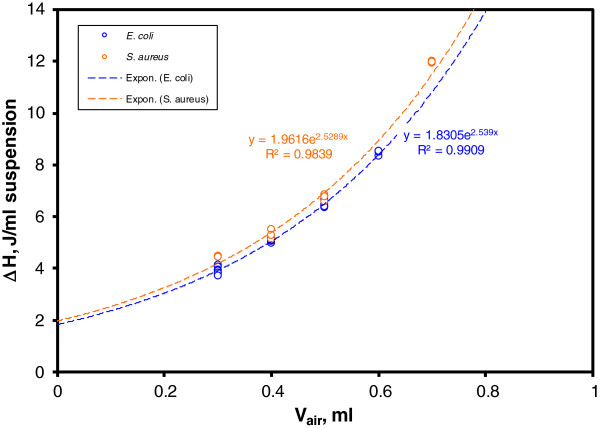
**Specific total thermal growth ΔH (J/ml) variation with the air volume content of the cell, calculated as (1 - V**_**sample**_**) ml.** The exponentially fitted graphs of *Escherichia coli* and *Staphylococcus aureus* are quite similar, despite the marked differences in their respective thermograms.

Overall heats (total thermal growth) for the 18 *E. coli* runs and 8 *S. aureus* runs are plotted in Figure 2 against the air volume contained in the measuring cell, evaluated as [1 – sample volume (ml)] (1 ml is the nominal batch cell volume). There is an obvious overlap of the dependence of specific total heat ΔH (J/ml suspension) for the two strains, despite of the above-mentioned qualitative differences in the corresponding thermograms. Due to the fact that all runs involved the same initial bacterial concentration, we can conclude that for the investigated bacterial strains the overall thermal growth effect is not strain dependent, but rather air volume dependent. The exponential fits of the two strains, presented in Figure 2, are quite similar.

#### Differences

**Figure 3:**
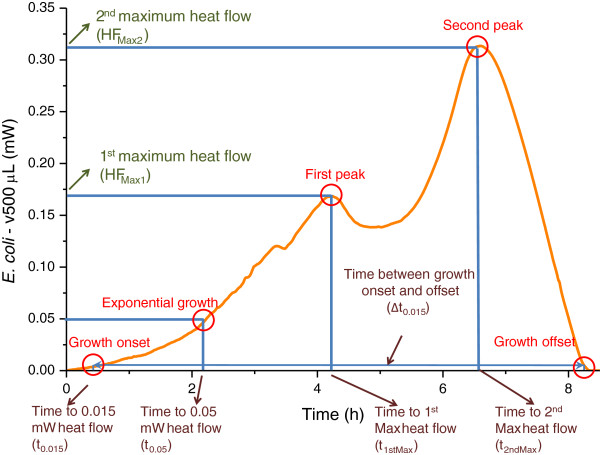
**Graphical representation of the proposed 5 points of interest that could be utilized as thermal growth characteristics of the two strains.** The parameters and nomenclature proposed for the statistical evaluation of bacterial thermal growth.

A set of quantitative parameters based on some key points of the thermogram was proposed and analyzed. These points are: thermal signal detection, establishment of the exponential growth, the first peak maximum, the second peak maximum and the return to baseline. Associated quantities to these points (times, i.e. corresponding positions or intervals on the time scale and heat flow values) can be used to characterize raw bacterial growth thermograms as well as to differentiate the two bacterial strains (Figure 3, Table 1). For growth detection, other investigators have chosen a threshold value of the recorded heat flow of 0.01 mW [15]. A value of 0.015 mW was chosen in the present analysis for both bacterial growth detection and return to baseline (onset and offset of thermal growth). “t_0.015_” corresponds to the time needed to reach this value and “Δt_0.015_” corresponds to the time difference between offset and onset (growth detection and return to baseline). This is well above the μDSC sensitivity and noise threshold (μDSC3 – 30 nW, μDSC7 – 20 nW, according to manufacturer’s specifications) and corresponds to 5-15 × 10^6^ thermally active bacteria [1]. The value of 0.05 mW was chosen for the exponential growth (“t_0.05_” is the time needed to reach this heat flow value) as this value lies within the time period of fully established exponential growth regime for both strains. It corresponds to the thermal activity of 2-5 × 10^7^ bacteria.

**Table 1:** Proposed bacterial microcalorimetric growth parameters for characterizing a raw thermogram

**Parameter**	**Description**
t_0.015_ (h)	Time to 0.015 mW heat flow, i.e. thermal growth onset time
t_0.05_ (h)	Time to 0.05 mW heat flow, i.e. established exponential growth time
t_1stMax_ (h)	Time to 1^st^ maximum heat flow, i.e. time to first peak
t_2ndMax_ (h)	Time to 2^nd^ maximum heat flow, i.e. time to second peak
Δt_0.015_ (h)	Time between thermal growth onset and offset
HF_Max1_ (mW)	First maximum heat flow, i.e. first peak amplitude
HF_Max2_ (mW)	Second maximum heat flow, i.e. second peak amplitude

#### Data analysis on raw (non-normalized) thermograms

All thermograms were processed as previously described [7,16,17] with baseline and time correction, thus eliminating the initial thermal perturbations and adjusting all experiments to a zero time reference. The baseline was calculated and subsequently subtracted using either Calisto software v1.077 (AKTS) and/or Peakfit v4.12 (SYSTAT). Zero time correction was done in Peakfit using data exported in Excel from Calisto; the final plots were done using the OriginLab Origin v. 8.1 and the Microsoft Excel software. For the statistical analysis we used SPSS 16.0 software (SPSS, Inc, Chicago, Illinois). Data from 18 runs performed on *E. coli* and 8 on *S. aureus* with sample sizes of different volumes were analyzed, as shown in Figure 1. One may easily notice significant qualitative differences between the 2 strains. The Shapiro-Wilk [18] validity test performed on the 2 sets of data indicated a normal distribution for all parameters of *E. coli* and for 4 out of 7 of *S. aureus* thermal growth (t_0.015_, t_0.05_, Δt_0.015_, HF_Max1_). Results are expressed as mean and standard deviation for normally distributed continuous variables (further analyzed by Student *t* test), or median and minimum/maximum for non-normally distributed variables (analyzed by Mann–Whitney *U* test). Hypothesis testing was 2-tailed, with P < 0.05 considered statistically significant. The statistical independent *t*-test [19] (CI = 95%, α = 0.05) and the Mann–Whitney *U* test performed on the 7 parameters proved that there is a statistically significant difference (with a p value < 0.0001) between the two strains (Table 2). This difference is not volume-dependent, since all the experiments were introduced in the statistical analysis, regardless of the bacterial culture volume used.

**Table 2:** **Statistical analysis (*t*-test and Mann–Whitney U) results for strain differentiation on raw data; time (hours); heat flow (mW)**

**Parameter**	***Escherichia coli***	***Staphylococcus aureus***	**p value**	**AUROC**
	**Mean (SD)**	**Mean (SD)**		
	**median (min, max)**	**median (min, max)**		
t_0.015_ (h)	0.7733 (0.31410)	1.5244 (0.35735)	< 0.001*	0.979
t_0.05_ (h)	1.6786 (0.46648)	2.9969 (0.53285)	< 0.001*	0979
t_1stMax_ (h)	3.92 (2.75, 4.59)	5.27 (4.08, 5.59)	0.002**	0.965
t_2ndMax_ (h)	6.35 (5.42, 7.11)	19.50 (14.19, 21.37)	< 0.001**	1
Δt_0.015_ (h)	6.38 (0.4719)	22.0963 (2.1973)	< 0.001*	1
HF_Max1_ (mW)	0.1937 (0.02234)	0.0859 (0.01214)	< 0.001*	1
HF_Max2_ (mW)	0.2126 (0.1, 0.31)	0.0306 (0.03, 0.04)	< 0.001**	1

*t (Student) test; **Mann–Whitney *U* test.

Among the 7 proposed parameters, some could be less reliable in practice, for different reasons:

– t_0.015_ (time to reach 0.015 mW heat flow, i.e. thermal growth onset time) is likely to be affected by signal perturbations at the beginning of the thermal run. Although this parameter offers the advantage of a faster result, it also bears the disadvantage of a lower difference in heat flow between strains. Even so, the differences between values of this parameter for the two investigated strains were proven statistically significant.

– The second maximum heat flow is more difficult to identify for *S. aureus*, thus the parameters t_2ndMax_ (time to reach the second maximum) and the HF_Max2_ (second heat flow maximum value) are less reliable.

– Δt_0.015_ (time between thermal growth onset and offset) offers the advantage of large differences between the 2 strains, but also the shortcoming of a late result (more than 10 to 12 hours).

Thus, the most convenient parameters among the 7 proposed for bacterial discrimination appear to be: t_0.05_ (1.67 ± 0.46 h for *E. coli* vs. 2.99 ± 0.53 h for *S. aureus*, p <0.0001), t_1stMax_ (3.92 (2.75, 4.59) h for *E. coli* vs. 5.27 (4.08, 5.59) h for *S. aureus*, p = 0.002) and HF_Max1_ (0.19 ± 0.02 mW for *E. coli* vs. 0.086 ± 0.012 mW for *S. aureus*, p < 0.0001).

By means of t_0.05_ one should be able to differentiate between strains in the first 3 to 4 hours of the experiment. Using the other 2 most reliable parameters related to the first heat flow maximum, one could differentiate strains in 5 to 6 hours; a high probability of discrimination results from the concomitant utilization of the three parameters. Thus, these parameters may be used in differentiating between *E. coli* and *S. aureus.* A reasonable extension of this approach points to the construction of bacterial microcalorimetric databases in well-defined growth conditions.

#### Data analysis on volume-normalized thermograms

To reduce the influence of sample volume on statistical data, volume-normalized thermograms were generated in Calisto and are presented in Figure 1b. Based on considerations similar to the ones used for the raw signals, 4 parameters were proposed and analyzed for volume-normalized thermograms (see Table 3). These are related to the first peak of the normalized thermogram because this peak appears to be less influenced by the air volume present in the cell (see *infra* – oxygen dependence of growth).

**Table 3:** Proposed bacterial microcalorimetric growth parameters for characterizing a volume-normalized thermogram

**Parameter**	**Description**
tn_0.05_ (h)	Time to reach a sample volume normalized heat flow of 0.05 mW/ml
tn_0.1_ (h)	Time to reach a sample volume normalized heat flow of 0.1 mW/ml
tn_Max1_ (h)	Time to reach the 1^st^ peak maximum
HFn_Max1_ (mW/ml)	First peak amplitude (sample volume normalized heat flow)

The Shapiro-Wilk data validity test indicated the validity of all parameters except for the first maximum of the normalized heat flow of *E. coli*. The statistical *t*-test (CI = 95%, α = 0.05) and the Mann–Whitney *U* test performed on the 4 parameters proved that there is a statistically significant difference (with a p value < 0.005) (Table 4). The most valuable parameters for bacterial differentiation using normalized thermograms seem to be tn_0.1_ (1.75 ± 0.37 h for *E. coli* vs. 2.87 ± 0.65 h for *S. aureus*, p <0.005), tn_Max1_ (3.78 ± 0.47 h vs. 5.12 ± 0.52 h, p < 0.0001) and HFn_Max1_ (0.33 (0.29, 0.47) mW/ml vs. 0.18 (0.13, 0.23) mW/ml, p < 0.001).

**Table 4:** **Statistical analysis (*t*-test and Mann–Whitney U) results for strains differentiation on normalized data; time (hours); normalized heat flow (mW/ml)**

**Parameter**	***Escherichia coli***	***Staphylococcus aureus***	**P value**	**AUROC**
	**mean (SD)**	**Mean (SD)**		
	**median (min, max)**	**median (min, max)**		
tn_0.05_	1.1505 (0.3557)	1.9206 (0.5063)	<0.001*	0.917
tn_0.1_	1.7489 (0.3742)	2.8718 (0.6471)	<0.005*	0.986
tn_Max1_	3.7819 (0.4671)	5.1243 (0.5236)	<0.001*	0.951
HFn_Max1_	0.33 (0.29, 0.47)	0.18 (0.13, 0.23)	<0.001	1

*t (Student) test; **Mann–Whitney *U* test.

Again, tn_0.1_ parameter could be used to differentiate between strains in the first 3 to 4 hours and the combination with tn_Max1_ and HFn_Max1_ parameters could be used with a very high probability to differentiate between strains in the first 5 to 6 hours. The slight differences regarding the statistical results regarding the time to reach the first maximum in non-normalized and normalized thermograms are caused by manual baseline corrections.

#### Statistical data analysis conclusions

Analysis of the proposed parameters display statistically significant differences between the 2 strains (p < 0.05). Moreover, the AUROC [20] (area under receiver operating characteristic) curves display high values (between 0.9 and 1) of all proposed parameters, which makes these parameters highly sensitive and specific in discriminating between *E. coli* and *S. aureus*. Within the range used in the present study (0.3 to 0.7 ml), the sample volume does not influence the discriminating power of the parameters explored (the time shifts were negligible). However, for practical microcalorimetric discrimination of different, unknown bacterial cultures, a crucial parameter to be rigorously controlled is the initial bacterial concentration (inoculum). The presented statistical analysis indicates a reasonable turbidity control of the inoculum, at least within the utilized experimental set.

An alternative approach consists in taking, e.g., t_0.015_ as zero reference time for samples of different initial concentration (inoculum size) that would mimic the hospital lab conditions. The thermal growth variability with inoculum size was explored in our previous contribution [7] involving freshly prepared inocula of *S. epidermidis* growth evaluated on the Setaram MicroDSC III. There are advantages and drawbacks to both sides of the dilution scale: diluted samples exhibit clear baselines at the beginning of growth, with time – extended thermograms; concentrated samples display time – compressed thermograms, the onsets of which are overlapping with the instrument equilibration (the growth starts before the instrument is ready to effectively measure it). As detailed in Methods, a compromise between the two situations was adopted within the present study, involving samples kept in cold storage (“dormant cultures”) of approximately the same initial concentration (turbidity controlled).

### In-depth analysis of the influence of experimental conditions on the bacterial growth thermograms

#### Oxygen dependence of growth

The oxygen content clearly influences the thermograms of both strains in different ways, probably due to different metabolic pathways (Figure 1). For *Staphylococcus aureus*, higher volumes of oxygen result in extended times of growth (broadening) associated with the second peak, while the effect on its height is less evident. For *Escherichia coli* the increase in air volume results in the increase of the height of the second peak that makes it a good predictor of the volume of available oxygen. The hermetical sealing of the microcalorimetric batch cells affords the estimation of the oxygen content influence on the growth of the two microorganisms. Due to different growth conditions, reported shapes of the thermograms pertaining to the same strain are often different. Out of several factors that contribute to the shape of the thermogram, the following analysis is restricted to the contribution of the oxygen (air) volume. As shown in Figure 2, samples with lower volumes produce higher amounts of heat per ml suspension. The most probable cause of this thermal effect increase is due to the larger amounts of oxygen available in the microcalorimetric cell headspace and, via diffusion, to bacterial growth.

#### Peakfit decomposition of the thermograms

A natural extension of the analysis is to decompose the observed thermal signal into its components (by means of Peakfit® - Systat software) and examine their variation with (cell headspace) air volume. [The term “deconvolution” is often improperly used for various cases of complex signal analysis. The confusion is due to the fact that any measured signal is indeed a convolution between the intrinsic contribution of the investigated physical-chemical process and the instrumental contribution. The two may be separated, i.e. deconvolved, by means of Fourier analysis, as in X-ray diffraction line broadening analysis. For most of the spectroscopy, chromatography and (micro)calorimetry data, the observed complex signal is a superposition (in fact, the sum) of various components (processes) that may be evidenced via Peakfit. Therefore, the term “decomposition” is the correct choice]. Growth patterns are clearly more complex, but as a first-order approximation, the two-peak decomposition was chosen, as described in Methods section. Prior to Peakfit decomposition all thermograms were normalized to the overall area, with the introduction of the “normalized heat flow”, NHF(t).

The main thermal quantities that can be obtained from the raw thermograms and their corresponding terms [8] inspired from Monod’s seminal contribution to bacterial growth [14] are given in Eq. (1):

(1) $$\begin{array}{l} \mathrm{HF}\left(t\right)=\mathrm{heat}\;\mathrm{flow}\;;\;\mathrm{\Delta H}(t)=\int_{t_{0}}^{t}\mathrm{HF}\left(t\right)\mathrm{dt}="\mathrm{thermal}\;\mathrm{growth}"\;\\ \Delta H_{\mathrm{tot}}=\int_{t_{0}}^{t_{\max}}\mathrm{HF}\left(t\right)\mathrm{dt}="\mathrm{total}\;\mathrm{thermal}\;\mathrm{growth}"\\ \mathrm{NHF}\left(t\right)=\frac{\mathrm{HF}\left(t\right)}{\Delta H_{\mathrm{tot}}}="\mathrm{normalized}\;\mathrm{heat}\;\mathrm{flow}";\langle \mathrm{NHF}\rangle ={\left(\mathrm{time}\right)}^{-1}\\ \mathrm{SHF}\left(t\right)=\frac{\mathrm{HF}\left(t\right)}{\mathrm{\Delta H}\left(t\right)}="\mathrm{specific}\;\mathrm{heat}\;\mathrm{flow}";\;\langle \mathrm{SHF}\rangle ={\left(\mathrm{time}\right)}^{-1}\\ \;\alpha \left(t\right)≜\frac{\mathrm{\Delta H}\left(t\right)}{\Delta H_{\mathrm{tot}}}=\mathrm{fractional}\;\mathrm{conversion};\;\frac{\mathrm{d\alpha}\left(t\right)}{\mathrm{dt}}=\mathrm{NHF}(t)\; \end{array}$$

A general feature of differential scanning calorimetry (DSC) signal is asymmetry [12,13]. Its major source is the non-isothermal nature of most DSC experiments, in which constant rate heating/cooling acts as the effluent in chromatography. For isothermal runs, such as microcalorimetric bacterial growth ones, no sizable instrumental contribution to the observed shape is expected: broadening (width) and asymmetry (fronting and/or tailing) are most probably caused by the complexity of the thermally measurable processes involved. Thus all fitting parameters of utilized functions were allowed to vary among the two peak components. Although some of built-in Peakfit functions rely on certain physical models for, e.g. chromatography experiments, all functions were strictly used as empirical means to decompose the observed thermal signal. HVL (Haarhof – Van der Linde) chromatography function was found as the most appropriate one in the description of microcalorimetric growth data:

(2) $$\mathrm{NHF}\left(t\right)\;=\;\frac{\frac{a_{0}a_{2}}{a_{1}a_{3}\sqrt{2\pi }}\exp\left(-\frac{1}{2}{\left(\frac{t-a_{1}}{a_{2}}\right)}^{2}\right)\;}{\frac{1}{\exp\left(\frac{a_{1}a_{3}}{a_{2}^{2}}-1\right)}+\frac{1}{2}\left[1+\mathrm{erf}\left(\frac{t-a_{1}}{\sqrt{2}a_{2}}\right)\right]}$$

In Eq. (2), fitting parameters have the following meaning: *a*_*0*_ = area, *a*_*1*_ = center, *a*_*2*_ = width (>0) and *a*_*3*_ = distortion, i.e. asymmetry (≠ 0). As data submitted to Peakfit decomposition involved area normalized thermograms, parameter *a*_*0*_ represents the fraction of the corresponding peak to the total thermal growth.

**Figure 4:**
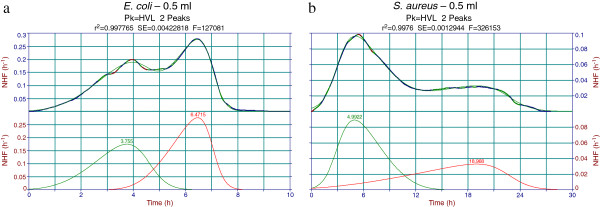
**Peakfit decomposition of *Escherichia coli *and *Staphylococcus aureus *normalized heat flow (NHF) average thermograms.** Two peak decomposition of average thermograms of 0.5 ml volume samples using the built-in Haarhof – Van der Linde (HVL) chromatography function. The two peaks may represent bacterial growth on behalf of dissolved (first peak) and diffused (second peak) oxygen. **a**. Fronted-fronted coupling for the *E. coli* thermogram decomposition. **b**. Tailed-fronted coupling for the *S. aureus* thermogram decomposition.

**Figure 5:**
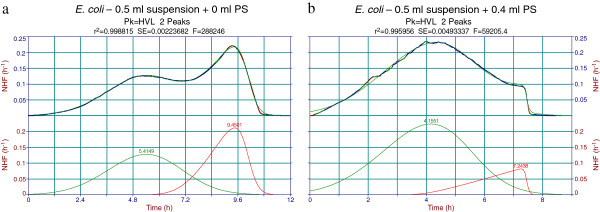
**Physiological saline (PS) dilution effect on Peakfit decomposition of *Escherichia coli *normalized heat flow (NHF) thermograms. a**. Two peak decomposition (HVL) of a normal 0.5 ml *Escherichia coli* thermogram (0 ml PS added, ~0.5 ml air volume). **b**. Two peak decomposition (HVL) of 0.5 ml *Escherichia coli* + 0.4 ml PS (~0.1 ml air volume).

**Figure 6:**
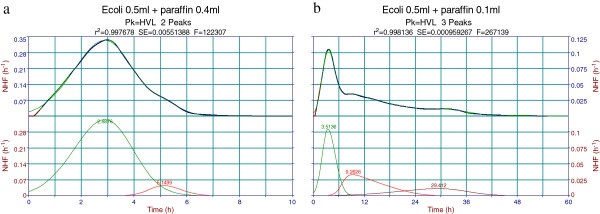
**Peakfit decomposition of *Escherichia coli *normalized heat flow (NHF) thermograms with oxygen diffusion suppression by mineral oil (MO). a**. Two peak decomposition of 0.5 ml *Escherichia coli* + 0.4 ml MO thermogram (~0.1 ml air volume). **b**. Three peak decomposition of 0.5 ml *Escherichia coli* thermogram + 0.1 ml MO (~0.4 ml air volume).

Figures 4, 5 and 6 contain examples of Peakfit analysis of experimental data. Figure 4 displays 2-peak decomposition of average thermograms pertaining to 0.5 ml samples of the two strains investigated. One may notice the fronted – fronted coupling for *E. coli*, whereas for *S. aureus* there is a tailed – fronted coupling. For other sample volumes peak 1 may change to a tailed shape but peak 2 retains its fronted shape for both strains. There is a monotonous decrease of peak 1 and increase of peak 2 with decreasing of the sample volume (which means increasing of the air - filled volume of cell headspace). This behavior is not conserved when working with other Peakfit library asymmetric functions (EMG, GMG, LogN, etc.) and this was one of the main reasons for the selection of the HVL function. Indeed, with the non-negligible noise of the microcalorimetric data, and with the unlocked (freely varying) fitting parameters, the software automatically selects the best possible fit in statistical terms (F-statistic, standard error, correlation coefficient). A consistent variation of the fitting parameters with the variation of some experimental factor (sample or air volume) is therefore a bonus to seek for, and that was found in the case of HVL function.

Complex thermal growth patterns, called “biphasic thermograms”, were previously reported for the calorimetrically investigated metabolism of yeasts [21]. They were attributed to a shift in the carbon source for culture media consisting of mixtures of mono and disaccharides or different disaccharides and discussed in terms of “constitutive and inducible transport systems and degradation enzymes”. The reported results were considered as the thermal expression of the phenomenon termed by Monod “diauxie” [22]. Double-peak thermograms were also ascribed to “anaerobic – aerobic growth” [23]. Proof of the actual aerobic growth of *E. coli* K-12 at nano-molar oxygen concentrations has been recently presented [24]. Attempts of more detailed descriptions have been made, with no further development of the argument or an in-depth investigation [1]. The closed batch cell experimental conditions used within the present study are different from either continuous, oxygen concentration controlled flow [24] experiments, or “N_2_ fumigated” [2] (i.e. flushed suspension) batch ones. This is clearly not the case within the present experimental setup, where one may acknowledge the existence of suspension pre-dissolved oxygen and available headspace gaseous oxygen able to diffuse in the bacterial suspension. The culture medium utilized is a nutrient - rich one, containing a sufficient amount of glucose: a shift in the carbon source resulting in diauxic growth is therefore less probable within the experimental setup utilized in the present study. Moreover, supplementary physiological saline dilution and mineral oil addition experiments, described below, point to a different interpretation.

The natural approximation of the complex processes that take place inside the o-ring sealed batch cell is that oxygen is a limiting thermal growth factor (terminal electron acceptor): the first process (peak) may be ascribed to “dissolved oxygen growth” and the second one to “diffused oxygen growth”. To support the assumption that the second peak is indeed a diffused oxygen dependent process, additional experiments involving the decrease of the available air volume were performed with the *E. coli* strain.

- The first set involved progressive dilutions (0.1, 0.2, 0.3, 0.4 ml) with physiological saline (PS) of the same bacterial suspension sample of 0.5 ml. Figure 5 displays the dilution effect, as manifested in Peakfit decomposition of the initial (0.5 + 0 ml) and most diluted (0.5 + 0.4 ml) samples. One may readily observe that while the first peak shape is similar, the second one is clearly affected. With the normalized heat flow representation of the thermogram, the weights of the two peaks display the expectable opposite variation: peak 1 increases while peak 2 decreases with PS dilution. The nominal volume of the batch cell is 1 ml, but a complete filling with liquid suspension is not possible. The maximum sample volume achieved in dilution experiments was 0.9 ml. The still present gaseous oxygen in the cell headspace accounts for the observed thermogram and Peakfit decomposition: as the dissolved oxygen is consumed in the first process (peak), gaseous oxygen diffusion in the depleted suspension generates the second peak that accounts for a slower, diffusion-limited growth. Detailed quantitative analysis of the associated thermal effects (total and “peak” thermal growth) will be presented at the end of this section.

- An additional check of the gaseous oxygen influence on the observed growth patterns involved adding of sterile paraffin oil to the same 0.5 ml sample of *E. coli*. In principle, this should inhibit oxygen diffusion and thus peak 2. Figure 6 displays two experiments with (a) 0.4 ml oil and (b) 0.1 ml oil. The amount of 0.4 ml paraffin oil seems to be sufficient for an almost complete suppression of the second peak. Its presence, even severely diminished, may be due to either gaseous oxygen diffusion through the oil layer or transport of oil dissolved oxygen to the depleted bacterial suspension. Oxygen diffusion in paraffin oil at 37°C was claimed to reach about 2/3 of that in water at the same temperature [25]. This seems to explain the more complex growth behavior displayed in Figure 6b, where 0.1 ml mineral (paraffin) oil barrier is clearly penetrated by oxygen (present in the unfilled 0.4 ml headspace of the cell). The best decomposition of this extended (≈ 60 hours) experiment actually involves 3 peaks: the first one clearly pertains to “dissolved oxygen” growth; the second accounts for “mineral (paraffin) oil hindered diffused oxygen” growth; the third may be due to a fully fermentative growth switch of (some fraction of) the bacterial population.

#### Variations of total and peak thermal effects

“Thermal growths” associated to overall thermograms (total thermal growths) and to the corresponding components (peak or process thermal growth) were further analyzed. Total growth heats expressed as specific values (in J/ml suspension), or absolute values (in J) were calculated from raw thermograms in Calisto. The corresponding peak (growth process) values are simply obtained by multiplication with the *a*_*0*_ Peakfit parameter, which equals its (area) fraction to the overall effect.

**Figure 7:**
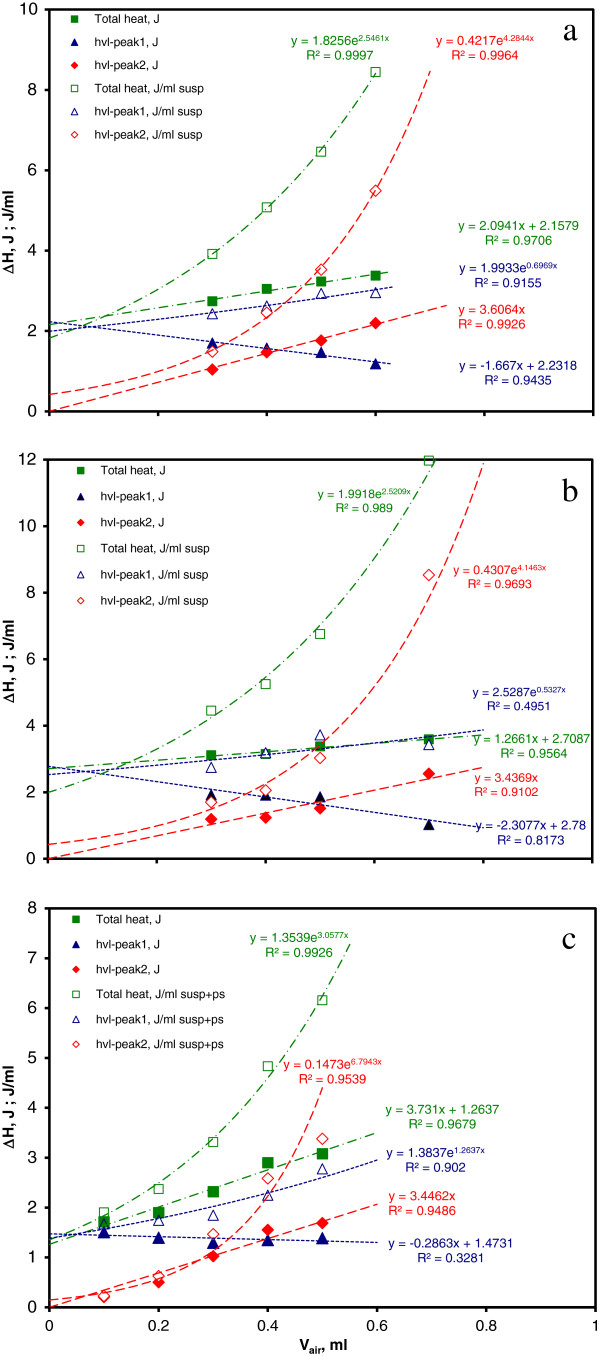
**Variation of the absolute (J) and specific (J/ml suspension) thermal effects with available air volume (ml). a**. Total and peak values for *Escherichia coli* average thermograms. **b**. Total and peak values for *Staphylococcus aureus* average thermograms. **c**. Physiological saline dilution values for *Escherichia coli* thermograms. Specific heats are fitted with exponential trendlines, while absolute heats are fitted with linear ones. “hvl-peak1” and “hvl-peak2” represent the contributions of the two Peakfit components to the overall thermal effect.

Variations of the heat effects with available air volume are presented in Figure 7, as follows: 7a average values for *E. coli* runs analyzed in Section B; 7b average values for *S. aureus* runs analyzed in Section B; 7c *E. coli* physiological saline dilution runs. As in Figure 3, specific total and peak heats (J/ml suspension) that display a non-linear variation with cell headspace air volume were fitted with exponentials. Average values were used in Figure 7a and b, whereas values for all runs are given in Figure 3: therefore, slight differences of the fitting parameters may be noticed. Absolute total and peak heats (J) display fairly linear variations with air volume (with better correlation for *E. coli* than *S. aureus*). For graphic purpose, “hvl-peak2, J” fits were forced to zero intercepts; actual values were slightly below, but close to zero (0.074 J for *E. coli*, 0.071 J for *S. aureus* and 0.21 J for *E. coli* dilution). This is consistent with the assumption of a diffused oxygen growth described by “hvl-peak2” that vanishes at zero air volume within the batch cell.

There are several other regularities in Figure 7 that support both the assumed dissolved – diffused oxygen growth interplay and the actual HVL Peakfit decomposition of the observed thermograms:

• Decrease of “hvl-peak2, J” and increase of “hvl-peak1, J”

• Intercepts of “hvl-peak1, J” and “Total heat, J” are, within experimental errors, practically identical. This reflects the hypothetical situation of a calorimetric cell completely filled with bacterial suspension: in this case the whole thermal growth is given only by dissolved oxygen, i.e. by peak 1.

• Fairly close to the above values are the intercepts of the exponentially fitted specific values “Total heat, J/g” and “hvl-peak1, J/ml suspension”. (*S. aureus* values are more scattered, reflecting the scatter of the pertaining raw thermograms). This is the expected behavior for a 1 ml nominal volume of the cell: for a completely filled cell absolute (J) and specific (J/ml) values of the thermal effect are supposed to coincide.

The results presented in Figures 4, 5, 6 and 7 consistently support the idea that complex thermal growth patterns as the ones obtained in the present contribution are mainly due to the interplay between dissolved and diffused oxygen. Truly fermentative growth is not excluded, but its thermal contribution seems to be of minor importance within the growth conditions utilized. The most probable metabolic pathway accounting for bacterial growth of *E. coli* in batch Hastelloy cells is an aerobic one, with dissolved and diffused oxygen acting as a growth limiting factor and resulting in the two-peak thermal growth thermograms.

#### Long term refrigeration viability counts check

**Figure 8:**
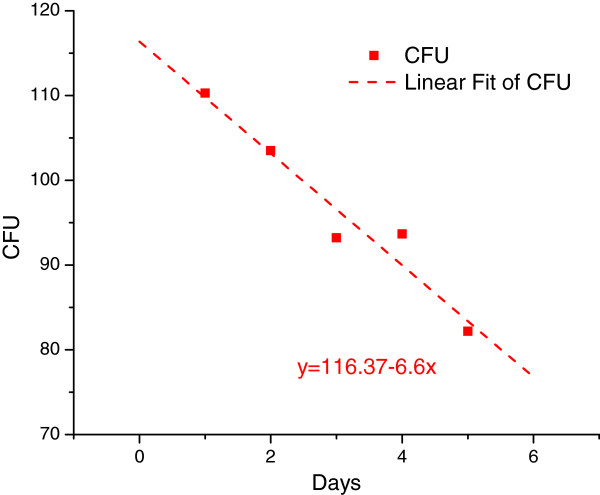
**Variation of viable counts (VC) with the time spent in cold storage.** The linear fit of the slight decrease of colony forming units (CFU) within 1 – 5 days spent in cold storage (4°C). VC pertain to samples stored in batch cells as detailed in Methods section.

As the two MicroDSC instruments utilized in the present study are single-channel, they can run one sample at a time. Microcalorimetry is very sensitive in detecting small variations in the bacterial density of the inoculum: this is fairly similar to the situation encountered in a busy clinical microbiology laboratory, where each new strain would require rapid processing and analysis. Under such circumstances, even the small variability that takes place in-between experiments needs to be assessed. A series of experiments was performed to evaluate the effect of refrigeration and long-term storage on the CFU viability count, as described in Methods section. Results are shown in Figure 8 where one may notice a fairly linear decline in CFU count with the time spent in cold storage. Some cells die during cold storage and this lowers the initial concentration of the sealed samples, resulting in longer growth time lags.

### Discrimination of bacteria based on local versus overall thermogram features

A most interesting approach to bacterial growth discrimination based on the thermal microcalorimetric signature was advanced by Bermúdez, López et al. more than 25 years ago [26,27]. Based on differences between unknown and “standard” (library stored) reference thermograms, the proposed method defines the “identification coefficient”, *I*, as an objective criterion of discrimination between bacterial species and even strains. *I* coefficient is the product *I = C*_*c*_ *× C*_*E*_ *× C*_*M*_ *× C*_*R*_, where *C*_*c*_ is the cross-correlation coefficient pertaining to whole thermograms, termed “p-t curves”. Other factors, termed “specific coefficients”, pertain to different parameters of the thermogram: *E* is “a measurement of the total energy dissipated by the culture during its growth”; *M* is “the maximum value of the dissipated power”; *R*, “the maximum metabolic rate”, is the maximum value of the time-derivative of the heat flow. The initial approach [26] was further developed [27] with the inclusion of the thermogram time-derivative, called “t-d curve” into a more complex “discriminant analysis” that was able to objectively evidence differences between strain growth patterns. One may easily notice the equivalence of some of the above parameters with quantities utilized in the present paper: *E ↔ ΔH*_*tot*_ and *M ↔ HF*_*max*_, respectively. There is another natural similarity between the two approaches which involves the well-defined growth conditions, a normal requirement for comparing the growth of different cultures. Besides the differences in statistical/mathematical processing, one may outline several differences between the two methods.

One may use the term “overall” for the method of Bermúdez, López et al., with a double-meaning: (i) the whole growth thermogram is needed for all key quantities *C*_*c*_*, C*_*E*_*, C*_*M*_*, C*_*R*_; (ii) the raw thermal signal, consisting of several overlapping metabolic processes is subject to statistical analysis. In fact, the authors seek for maximum complexity of growth (by adjusting the culture medium) as a necessary condition for discrimination between species. The present study involves both “overall” and “local” aspects: (i)’ the whole thermogram is needed for decomposition and *ΔH*_*tot*_ evaluation; (ii)’ discrimination parameters are looked for in component (local) features of the thermogram, with some (possible) metabolic significance.

The present study may be regarded as a start for further, extended investigations for other species and strains. Optimization of the advanced procedure for different thermal data is straightforward. As obtaining of sufficient data is time-consuming with single-channel microcalorimeters, the presented analysis was intended to avoid Lamprecht’s [28] caveat: “In our high-tech time of stream-lined instruments with black-box character, we experience automatic inputs, outputs, and computer calculations that do not allow getting to the roots of the thermal data”.

## Conclusions

Bacterial populations of *Staphylococcus aureus* and *Escherichia coli* exhibit different microcalorimetric growth patterns in both qualitative and quantitative assessments. The devised experimental routine (based on thermograms obtained from samples kept in cold storage, sealed in the measuring batch cells [7]) is sufficiently reproducible and accurate. The use of the 3 most reliable parameters defined for both raw and volume-normalized thermograms (t_0.05,_ t_1stMax_, HF_Max1,_ tn_0.1,_ tn_Max1,_ HFn_Max1_) offers a high probability of discrimination between the 2 strains within the first 5 to 6 hours of growth. The first parameter (t_0.05,_ tn_0.1_) offers a good probability of discrimination between the two strains within the first 3 to 4 hours of the growth process.

The discrimination method advanced in the present contribution has its limitations. The assumption that it can be used for *S. aureus* and *E. coli* needs extended research to be applied to other bacterial strains. For samples with same initial bacterial concentration but different volumes variability encountered within the same strain is smaller than the differences between the studied strains, allowing for discrimination. Variation of the initial bacterial concentration also requires supplementary investigation, as this is known to markedly influence the growth time lag and thus the proposed time parameters. As microcalorimetric data on bacterial growth is accumulating, interest in this method is expected to result in standardization of the optimal bacterial concentration and sample volume involving different research centers. For the time being, this method is not intended to be used in clinical practice with raw biological products (sputum, blood) as there is no control on bacterial sample concentration and other cell populations that could contaminate the thermogram.

Extension of the microcalorimetric growth pattern characteristics to other bacterial populations, with the eventual build-up of a database, may prove to be sufficiently accurate for bacterial strains discrimination. The information presented within this contribution may complement recent attempts to evaluate antimicrobial [5,6,29-31], antiparasitic [32], or antifungal [33] action on microcalorimetry monitored growth of various strains.

Peakfit decomposition of the thermograms obtained within specified conditions of this study and the quantitative analysis of thermal effects advanced herein point to an oxygen-controlled bacterial growth, at least in its thermal manifestation. There is an interplay between dissolved and cell headspace diffused oxygen: their contribution to the observed thermal behavior may be accounted for in terms of Peakfit decomposition of the overall thermogram. The advanced approach may offer solutions for deeper insight into bacterial metabolism, for the application of various bacterial growth models as well as for recently raised issues of “flask-to-medium ratio in microbiology” [34]. A systematic Peakfit analysis of such complex thermal growth patterns seems to be mandatory for the determination of the optimal growth conditions required for standardization and essential for the extensive use of microcalorimetry in clinical applications.

## Methods

### Microcalorimetry

Two Setaram Differential Scanning Microcalorimeters (MicroDSC) were used in the present study: the MicroDSC III and MicroDSC VII Evo. Both instruments were Joule effect factory calibrated and periodically checked with the factory naphthalene standard. Each calorimeter had an outer thermostatic loop provided by a Julabo F32-HE device operating in standard mode. 3D sensor protection was provided by a Nitrogen gas purge (99.99% SIAD - TP). The Calisto v1.077 software package was used for data acquisition and primary signal processing. This included baseline integration end export in Excel with equally spaced time increments. Heat values obtained were further analyzed in Excel and Origin 8.1. Exported baselines were further processed in Peakfit.

### Peakfit processing of the thermograms

Data exported from Calisto were processed in Peakfit by means of previously reported routines [16,17]. Whenever necessary, Savitsky-Golay smoothing was performed, generally with the “Al Expert” option. Calisto-generated baselines were imported and subtracted from the heat flow (HF, mW) signal. The time zero was changed for each thermogram by means of “Enter Calculation” option in Peakfit, allowing to a left shift of the whole data corresponding to the left intersection of the baseline and HF. This procedure brings all thermograms to a common X (time) scale, but definitely excludes any analysis of the growth lag time. The resulted data were subjected to “Area normalize” resulting in the “normalized heat flow” (NHF, h^-1^) [16,17]. This brings all thermograms to a common Y (NHF) scale, with the advantage that areas of the component peaks represent their fraction to the overall thermal effect. All subsequent peak fitting involved the NHF – time thermograms. Several built-in asymmetric peak functions were tried (EMG, GMG, LogN, Giddings, Pearson IV, HVL, etc.). The best one for the analyzed data proved to be the Haarhof – Van der Linde (HVL) chromatography function. This function resulted in both the best statistical criteria (r^2^, F-statistic, standard errors, etc.) and most reliable variations of the fitting parameters among the member of each set. As detailed in section C1, peak parameters were allowed to vary independently through the “Vary Widths” and “Vary Shape” options. The “Medium (Lorentz Err.) Robust Minimization” procedure was applied instead of the classical least-squares one.

### Bacterial strains

The reference strains of *Staphylococcus aureus* - *ATCC 25923* and *Escherichia coli* - *ATCC 25922* were used throughout the present study.

### Culture media

Bacterial culture media were prepared from stock Tryptic Soy Broth (TSB, Oxoid, UK), which is a mixture of Pancreatic digest of casein (17 g), NaCl (5 g), Papaic digest of soybean meal (3 g), K_2_HPO_4_ (2.5 g), Glucose (2.5 g) to 1 Liter and a pH of 7.3 ± 0.2 at 25°C. The medium was autoclaved before use and was microbiologically pure.

For viability counts, preparation of isolated colonies for inoculation and random sample check of aseptic technique, we used plates with Tryptic Soy Agar (TSA, Oxoid, UK); this solid medium has the same basic composition as TSB.

### Procedure

Each batch cell has a nominal capacity of 1 ml, and was filled with different volumes of inoculated TSB (sample volume varied from 300–700 μL), with the remaining free space being occupied by ambient air.

### Sample preparation

Before use, stock *Staphylococcus aureus* and *Escherichia coli* were streaked onto TSA plates. The baseline value of sterile TSB was recorded in McFarland Units with a Den-1 Nephelometer (Biosan, Lat). This value was subtracted from further measurements to obtain the true nephelometric value of the growing inoculum. Isolated colonies were picked-up with an inoculation loop and aseptically passed into a sterile tube containing 5 ml of TSB. This sample was grown until it reached a value of 0.5 McFarland units. 100 μL of this bacterial suspension were then transferred into a second nephelometric tube filled with 3 ml TSB and the resulting suspension was grown up to 0.1 McFarland. This suspension of the second tube was diluted a hundred fold and further used for μDSC runs.

### Microcalorimetric cell filling

The nominal volume of a batch calorimetric cell is 1 ml. However, in practice the maximum volume available for liquid sample filling for the o-ring sealed cell was 0.9 ml. The cell headspace air volume was calculated as (1 – V_sample_) ml for all runs. The experiments required three types of sample preparations:

1. Simple culture media samples

The microcalorimetric cells were filled with the required volume of sample at room temperature inside a laminar flow biosecurity hood and were hermetically sealed with their silicon o-ring covers. The time required to fill the cells was under 5 minutes, so significant thermogram differences are not expected to arise from the time needed to accomplish this procedure.

2. Physiological saline diluted samples

Physiological saline was added to the calorimetric cells filled with bacterial suspension, as described above.

3. Mineral oil (MO) covered samples

Sterile mineral (paraffin) oil (Sigma, DE) was carefully added at the air-fluid interface of the simple culture media sample, resulting in a three-phase sample: air, oil (meant as a barrier to oxygen diffusion) and bacterial culture.

### Experiments on samples kept in cold storage

A series of samples of the same turbidity, prepared as described above, were stored and kept for 1 to 5 days at 1-4°C. The experiments were performed at 1 day intervals using these samples.

### Viability counts

To correlate the number of starting viable bacteria with the microcalorimetric signal, some of the cells were filled with an excess of 100 μL sample. Before each microcalorimetric run, the cell content was thoroughly homogenized, and the excess sample was removed from the cell. The extracted 100 μL surplus was diluted a hundred fold and 50 μL was plated by dispersion onto TSA plates for CFU count.

### Microcalorimetric runs

The experiments were performed at 1 day intervals using samples kept in cold storage. The microcalorimeter was allowed to reach thermal equilibrium at 4°C for about 15 min. The sample cells were then taken out of cold storage and rapidly introduced in the calorimeter, allowing an extra hour for the calorimeter thermal stability at 4°C. Working temperature was reached by ramp heating with 0.5 K/min. In all experiments, the reference was a batch o-ring sealed cell containing an equivalent volume of:

1- Non-inoculated TSB,

2- PS-diluted non-inoculated TSB,

3- Sterile mineral oil + non-inoculated TSB, depending on the type of experiment.

## Abbreviations

TSA: Tryptic Soy Agar; TSB: Tryptic Soy Broth; McF: McFarland units; CFU: Colony Forming Units; E. coli: *Escherichia coli*; S. aureus: *Staphylococcus aureus*; MO: Mineral oil; PS: Physiological saline; HF: Heat flow; NHF: Normalized heat flow; IMC: Isothermal microcalorimeters; HVL: Haarhof, Van der Linde; GMG: Gaussian modified Gaussian; EMG: Exponentially modified Gaussian; LogN: Log Normal; μDSC: Differential scanning microcalorimetry; ATCC: American Type Culture Collection; VC: Viable counts.

## Competing interests

Financial competing interests

None of the authors of this contribution have any financial competing interests to report:

- None of the authors received in the past five years any reimbursements, fees, funding, or salary from an organization that may in any way gain or lose financially from the publication of this manuscript.

- None of the authors hold any stocks or shares in an organization that may in any way gain or lose financially from the publication of this manuscript.

- None of the authors hold or are currently applying for any patents relating to the content of the manuscript.

- None of the authors received reimbursements, fees, funding, or salary from an organization that holds or has applied for patents relating to the content of the manuscript.

- The authors have no other financial competing interests.

Non-financial competing interests

The authors don’t have any non-financial competing interests (political, personal, religious, ideological, academic, intellectual, commercial or any other) to declare in relation to this manuscript.

## Authors’ contributions

DCZ carried out bacterial cultures and inocula preparation; data acquisition and analysis and statistical data processing; AAM, ATS and OB carried out bacterial cultures and inocula preparation; MicroDSC experiments and data acquisition and analysis; MGP, RM, IT and CI carried out bacterial cultures and inocula preparation; VTP initiated and conceived this study, designed and supervised MicroDSC experiments and data analysis process, supervised the preparation of the manuscript; CB designed and performed the statistical analysis of the experimental data; MAB initiated and conceived this study; supervised the preparation of the manuscript; MIP initiated and conceived this study; designed and supervised bacterial growth; All authors participated in drafting of the manuscript and approved its final form.
